# Expression patterns of a circadian clock gene are associated with age-related polyethism in harvester ants, Pogonomyrmex occidentalis

**DOI:** 10.1186/1472-6785-9-7

**Published:** 2009-04-17

**Authors:** Krista K Ingram, Scott Krummey, Michelle LeRoux

**Affiliations:** 1 Department of Biology, Colgate University, Hamilton, New York, USA

## Abstract

**Background:**

Recent advances in sociogenomics allow for comparative analyses of molecular mechanisms regulating the development of social behavior. In eusocial insects, one key aspect of their sociality, the division of labor, has received the most attention. Age-related polyethism, a derived form of division of labor in ants and bees where colony tasks are allocated among distinct behavioral phenotypes, has traditionally been assumed to be a product of convergent evolution. Previous work has shown that the circadian clock is associated with the development of behavior and division of labor in honeybee societies. We cloned the ortholog of the clock gene, *period*, from a harvester ant (*Pogonomyrmex occidentalis*) and examined circadian rhythms and daily activity patterns in a species that represents an evolutionary origin of eusociality independent of the honeybee.

**Results:**

Using real time qPCR analyses, we determined that harvester ants have a daily cyclic expression of *period *and this rhythm is endogenous (free-running under dark-dark conditions). Cyclic expression of *period *is task-specific; foragers have strong daily fluctuations but nest workers inside the nest do not. These patterns correspond to differences in behavior as activity levels of foragers show a diurnal pattern while nest workers tend to exhibit continuous locomotor activity at lower levels. In addition, we found that foragers collected in the early fall (relative warm, long days) exhibit a delay in the nightly peak of *period *expression relative to foragers collected in the early spring (relative cold, short days).

**Conclusion:**

The association of *period *mRNA expression levels with harvester ant task behaviors suggests that the development of circadian rhythms is associated with the behavioral development of ants. Thus, the circadian clock pathway may represent a conserved 'genetic toolkit' that has facilitated the parallel evolution of age-related polyethism and task allocation in social insects.

## Background

Recent advances in sociogenomics, namely the ability to characterize molecular pathways in non-model organisms, provide novel opportunities to address questions concerning the evolution of social behavior in an ecological context by comparing taxonomically relevant species [1,2]. This evo-devo approach to behavior promises to be particularly successful in understanding the most extreme form of social organization, eusociality [3]. Eusocial species exhibit cooperative brood care, overlapping generations of individuals living together, and a division of labor in which one or few individuals monopolize reproduction. The Hymenoptera, including the ants, bees, and wasps, are ideally suited for comparative studies of social behavior as species in this group display a diversity of forms of social organization and represent multiple independent evolutionary origins of eusociality [4]. Research in the behavioral genetics of social insects reveals that molecular pathways associated with behaviors in social species include behavioral genes expressed in solitary species [reviewed in [5-9]]. To date, we have little information on whether genetic mechanisms are conserved across eusocial species or whether derived social phenotypes are a product of convergent evolution driven by alternative molecular pathways.

In a eusocial insect colony, behaviors must be coordinated or synchronized among potentially thousands of individuals at any point in time. In addition to a reproductive division of labor, non-reproductive workers in highly derived eusocial species also display a division of labor among adult workers who perform specialized colony tasks [4]. The success and survival of the colony depends on a variety of tasks; some tasks occur inside the nest, such as brood care and nest construction, while others occur primarily outside the nest, such as nest maintenance, nest defense, and foraging for food.

Task allocation, the coordination of behaviors in eusocial insect colonies, often occurs without any central or hierarchical command [10]. Individuals can display plasticity in their propensity to perform these behaviors throughout their lifetime [11]. In some highly derived eusocial species, individuals display age polyethism, i.e. they progress through sequences of several tasks in a development-dependent manner [1,4,12]. A recent review article by Toth and Robinson [1] highlighted comparisons between honeybee and harvester ants to illustrate potential scenarios explaining the independent evolution of age polyethism in eusocial insects. Age-related polyethism in ants and bees could either arise as a result of convergent evolution of behavioral phenotypes [1] or as a result of parallel evolution of behaviors via a conserved molecular mechanism shared by a common ancestral phenotype [1,13].

Efforts to determine the physiological and genetic bases for behavioral task development in non-reproductive eusocial workers have thus far focused on pathways involved in the development of nurse bees and foragers in honeybees [6,14-18]. One promising candidate pathway involves the role of the circadian clock in regulating age-dependent rhythmicity [14,16,19,20]. Honeybee foragers, which likely benefit from coordinating an internal clock with timing of nectar production and availability or sun-compass navigation, exhibit behavioral rhythmicity and a daily oscillation in the clock gene, *period *[14]. In contrast, nurse bees, which provide brood care inside the nest, exhibit arrhythmicity in locomotor behavior, and lower overall levels and weaker cyclical expression of *period *mRNA [14,16]. The results from honeybees suggest a link between foraging behavior, up-regulation of the *period *gene, and onset of circadian rhythms [14,16,20]. Given that age polyethism is a derived state in eusocial insect colonies [1,4], we ask whether foraging behavior is associated with the developmental regulation of circadian clock in the harvester ant, a species that evolved eusociality and age-related polyethism independently of bees [4,21].

The seed-eating harvester ants, genus *Pogonomyrmex*, are the behavioral model for task allocation in ants [1,10,12,22]. Large colonies of harvester ants (10,000–12,000 workers) inhabit the desert floors of the southwestern United States and the organization of these colonies runs, well, like clockwork. Young harvester ant workers tend to remain deep inside the nest with the single queen and perform tasks related to brood care, while older workers perform nest maintenance and patrolling tasks at the nest entrance and foraging tasks outside of the nest [12,23]. In *P. barbatus*, the species best characterized for behavior, foragers have specific behavioral patterns that relate to daily temperatures and environmental conditions [12]. In summer months, they emerge from the nest early in the morning and the majority of foraging occurs during the morning hours until the sun rises and temperatures increases. By late morning, most foraging activity has ceased, although some activity occurs again in the late afternoon. During the winter months, harvester ant colonies show little activity outside of the nest. Like honeybees, harvester ant foraging activity is dependent on light and temperature, but the developmental timeline of workers is vastly different between bees (0–20 days) and ants (months – year). Thus, we are interested in whether the association of foraging behavior and circadian rhythms is conserved across diverse developmental time scales.

We cloned a harvester ant ortholog to the *period *gene, *PoPer*, from a closely related species that is easily maintained in the laboratory, *Pogonomyrmex occidentalis*. We analyzed the expression of *PoPer *in brains isolated from individual workers in field-collected colonies housed under controlled light and temperature conditions in the laboratory. We show that *P. occidentalis *workers display a daily oscillation in *period *mRNA expressed in the brain and that this oscillation is endogenous. We compare the expression patterns of nest workers with foragers and show that differences in daily expression patterns correspond to differences in locomotor activity. In addition, we show that patterns of daily oscillations in *period *mRNA in foragers differ across seasons.

## Results

### *PoPer *expression in 12:12 LD cycle

**Figure 1:**
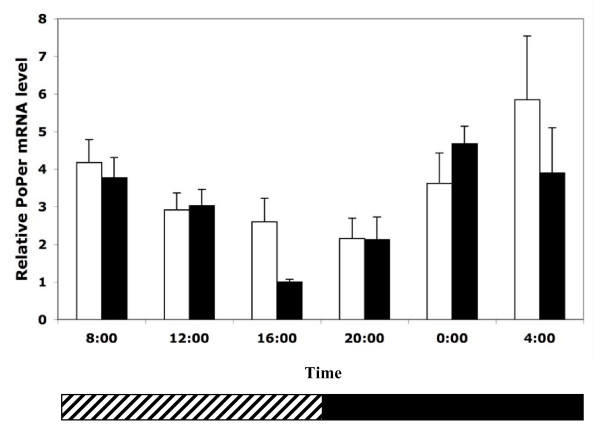
**Expression of *PoPer *mRNA in fall foragers in LD and DD conditions**. Relative expression of *period *mRNA in individual forager brains from experimental colonies of *P. occidentalis *collected in the early fall (w/SE). Colonies were entrained in controlled environmental chambers in either a 12:12 hr LD regime (light bars, n = 5 colonies) or a 24 hr DD regime (dark bars, n = 3 colonies). Bars represent the averages of colony means (four individuals sampled per colony at six time points for both light regimes; SE calculated from colonies). The diagonal stripes in the horizontal bar at base of the plot represent the actual (LD) or subjective light phase (DD) and the solid stripe represents the dark phase. All individuals were collected for analysis in dark conditions.

In the colonies collected in the fall (F06), foragers displayed a significant oscillation in *PoPer *mRNA expression with levels peaking in the late evening and the lowest levels occurring in late morning hours (Figure 1; F = 3.879, df = 5,11, p = 0.049).

**Figure 2:**
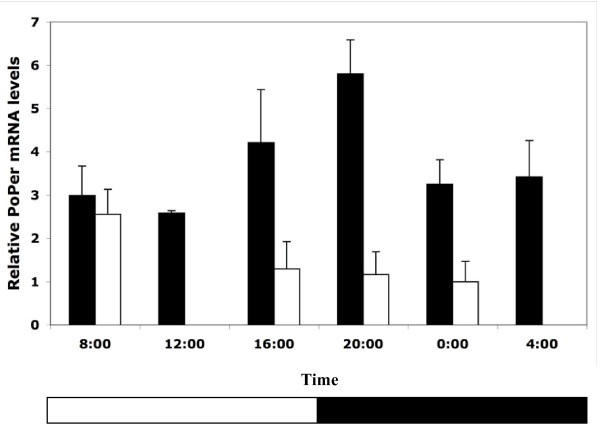
**Expression of *PoPer *mRNA in spring nestworkers and foragers (LD conditions)**. Relative expression of *period *mRNA in individual worker brains from experimental colonies of *P. occidentalis *collected in the spring (w/SE). Colonies were entrained in controlled environmental chambers in a 12:12 hr LD regime. Foragers were collected at six time points (dark bars, n = 4 colonies) and nest workers were collected at 4 time points (light bars, n = 4 colonies). Bars represent the averages of colony means with three nest workers and three foragers sampled per colony (SE calculated from colonies). The open stripe in the horizontal bar at base of the plot represents the light phase and the solid stripe represents the dark phase. All individuals were collected for analysis in dark conditions.

A similar pattern can be seen in foragers collected in the spring (S06) with the exception that there is a shift in maximum/minimum RNA levels (Figure 2; F = 3.519, df = 5,17, p = 0.023). In S06 colonies, the peak of mRNA expression occurs at least 4 hours earlier than the fall colonies and the lowest expression levels occur at 8:00 AM. Differences in *PoPer *mRNA levels across time points were not significant in spring nest workers (Figure 2; F = 1.453, df = 3, 11, p = 0.298), suggesting no oscillation in *period *expression levels in nest workers. Nest workers were found to have significantly different *PoPer *mRNA levels than foragers across time points (Table 1).

**Table 1:** Results from two-way ANOVAs on light condition and task.

**Foragers in LD vs. DD conditions (FALL)**			
	**df**	**F**	**Significance**
**Timepoint**	5	3.2	0.018*
**Condition**	1	0.497	0.485
Timepoint*Condition	5	0.589	0.709
			
**Nest Workers vs. Foragers in LD conditions (SPRING)**			

	**df**	**F**	**Significance**
**Timepoint**	5	1.2	0.350
**Task**	1	22.9	0.000*
Timepoint*Task	3	3.0	0.050*

*p-value ≤ 0.05. In F06, foragers were compared across two factors: time point and light regime (LD or DD condition). In S06, workers in LD conditions were compared across two factors: time point and task (nest worker or forager).

Although foragers from different colonies do vary in *PoPer *expression levels, no significant differences in expression patterns were observed among colonies across time points in F06 (F = 0.836, df = 5, 183, p = 0.559) or S06 (F = 1.042, df = 4, 172, p = 0.406).

### *PoPer *expression in 24 DD cycle

**Figure 3:**
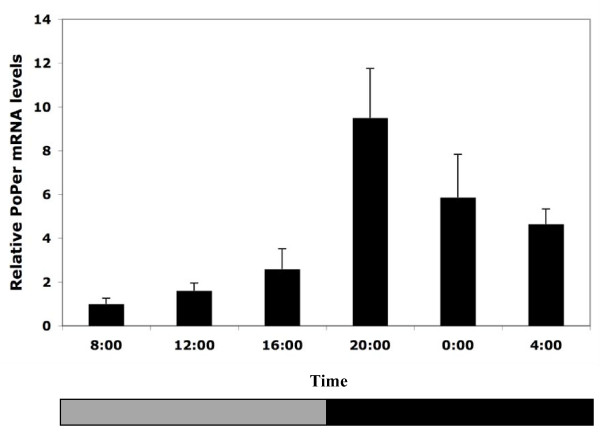
**Expression of *PoPer *mRNA in spring foragers (DD conditions)**. Relative expression of *period *mRNA in individual forager brains from three experimental colonies of *P. occidentalis *collected in the spring and entrained in a 24 hr DD regime (w/SE). Bars represent the averages of colony means with three foragers sampled per colony (SE calculated from colonies). The gray stripe in the horizontal bar at base of the plot represents the subjective light phase and the solid stripe represents the dark phase in DD conditions.

In DD conditions, *PoPer *mRNA expression levels were significantly different across time points in F06 foragers (Figure 1; F = 5.75, df = 5,67, p < 0.001). Patterns of expression were not significantly different between LD and DD F06 foragers (Table 1). A similar pattern occurs in the spring – S06 foragers in DD conditions have a significant increase in evening levels of *PoPer *mRNA (Figure 3; F = 7.32, df = 5,18, p = 0.0007). Overall differences in *PoPer *expression between 24 hr DD foragers and 12:12 hr LD foragers were not significant, but there was a significant interaction between time and light regime (F = 2.85, df = 5, 34, p = 0.0297).

### Behavioral analyses

**Figure 4:**
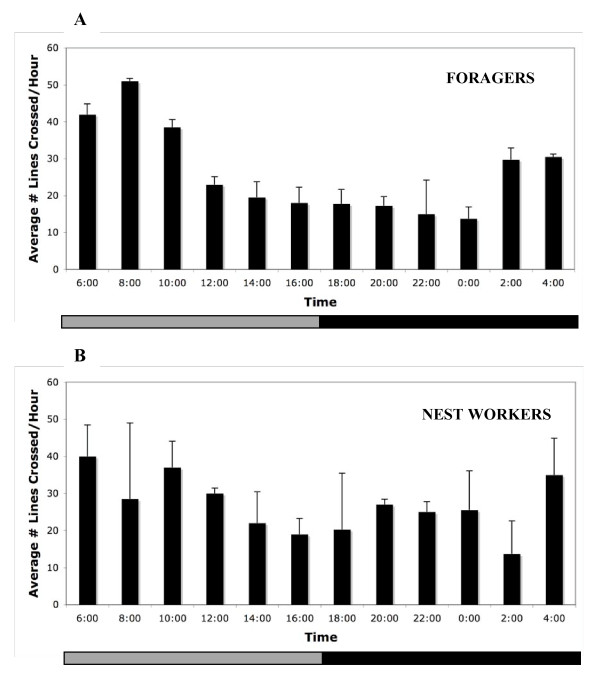
**Average locomotor activity of *P. occidentalis *workers in DD conditions**. Average levels of locomotor activity in individual *P. occidentalis *workers differ by task in DD conditions. Bars represent average values of locomotor activity in two-hour increments (w/SE, n = 5 foragers (A); n = 6 nest workers (B)). The gray stripe in the horizontal bar at base of the plot represents the subjective light phase and the solid stripe represents the dark phase in DD conditions. Forager activity levels (A) during the projected daytime were two times higher than activity levels in the projected nighttime. Nest workers (B) typically showed a brief peak of locomotor activity early in the projected morning and lower, variable levels of activity throughout the day and projected evening hours.

Forager activity levels during the projected daytime were two times greater than activity levels in the projected evening (Figure 4a). Nest workers typically showed a brief peak of locomotor activity early in the projected morning and lower, variable levels of activity throughout the day and projected evening hours (Figure 4b). Significant differences in activity levels were found between nest workers and foragers (F = 32.24, df = 1, 54, p < 0.0001) and across time points (F = 13.36, df = 5, 54, p < 0.001), and a significant interaction was found between worker task and time point (F = 20.36, df = 5, 54, p < 0.0001).

## Discussion

The daily behavioral rhythms of harvester ant foragers suggest a role of the circadian clock in mediating foraging behavior. Here we find that the expression pattern of *period *mRNA in forager brains is characterized by a strong diurnal fluctuation and that this oscillation is endogenous. *PoPer *mRNA is present in brains of young nest workers, but overall expression levels are lower than levels seen in foragers and there is no evidence of a diurnal cycle. Locomotor activity patterns of workers correspond with expression patterns – foragers show a diurnal cycle in activity levels while young nest workers show relatively continuous locomotor activity. Observed task-specific differences in *period *gene expression and behavioral activity levels suggest that nest workers are arrhythmic with respect to daily light regimes but foragers experience strong circadian rhythms in behavior. Our results indicate that the age-related division of labor in ants may be associated with both changes in the expression of a clock gene and the onset of behavioral circadian rhythmicity.

The association between oscillations in *period *expression and foraging behavior in ants parallels the pattern seen in honeybees. In bees, *period *expression levels are high in foragers relative to young nurse bees and strong, consistent oscillation in *per *mRNA levels are typically seen in foragers [14,16,20]. Nurse bees show greater variation in *per *levels, perhaps suggesting variation in developmental changes in anticipation of foraging behavior [20]. In harvester ants, young nest workers also had highly variable expression levels of *per *relative to foragers. Two alternative explanations exist for the absence of significant oscillation pattern in young harvester ant nest workers – difficulty in assaying significant changes in mRNA levels due to lower overall amounts of mRNA compared to foragers, or arrhythmicity among pooled individuals (the clocks of nest workers are operational, but not synchronized to other individuals). Due to the design of real-time PCR experiments on gene expression in brains, the sampling of individuals over time is not possible. However, the finding that the signature increase in forager *period *mRNA levels during the dark phase is not evident in the nest workers suggests that the diurnal fluctuations in *period *mRNA associated with clock function are not present in young ants. Thus, a similar mechanism of developmental regulation of a clock gene in social species may extend across different developmental time scales: bees develop from nurses into foragers in approximately 7–20 days, whereas ants develop into adult foragers over the course of months.

Both honeybees and harvester ants show higher overall levels of *period *mRNA in foragers, suggesting that there may be a functional significance to the increase of *per *during behavioral maturation. The difference between tasks is small (2–5 fold) but small changes in gene expression levels have been shown to have big effects on behavior, eg. [24]. In addition, recent work on suites of genes differentially expressed in honeybee nurse and worker tasks have revealed that many genes exhibit differences within this range [6,18]. It is not yet known whether the magnitude of differences in *per *mRNA levels translates into significant differences in Period protein in ants, but results from honeybees suggest that relative levels of Period protein correspond to daily fluctuations in *per *mRNA [25].

An important question is whether the circadian clock indeed functions to regulate behavioral maturation in social insects or whether the division of labor regulates the development of the circadian clock. Although the division of labor in ants and bees are associated with circadian plasticity and developmental regulation of *per *expression in ants and bees, it is difficult to directly infer functional causality because these traits are not measured in the same individuals. As molecular techniques in manipulating genomes of non-model organisms improve, direct tests of these hypotheses are necessary.

A related question that must be addressed is whether the development of clock function is associated directly with age, body size, social experience, or some other factor linked to task behavior. For example, circadian rhythms are associated with a size-dependent division of labor in bumblebees [26]. In honeybees, there is also evidence for reversals of behavioral rhythms in foragers that revert to brood care [27], suggesting that there is flexibility in systems of age polyethism [23]. In harvester ants, behavioral phenotypes are not limited to nest worker and forager tasks but include other specialized tasks both inside and outside of the nest [22]. Determining when the development of circadian rhythms and cyclic expression of *period *mRNA occurs in the intermediate tasks between young nest workers and old foragers will provide critical clues to understanding whether circadian rhythms are limited to foragers, involved in behavioral maturation of ants and/or due to behavioral experience outside of the nest.

Finally, our results suggest a link between seasonal variation in behavioral patterns of harvester ants and circadian clock gene expression. Circadian clock pathways respond to both light and temperature and it is thought that these two factors work in coordination to allow the clock mechanism to adapt to seasonal changes in photoperiod and temperature [28]. In *Drosophila, per *is rhythmic under light and temperature entrainment, but at low temperatures, there is a phase difference at transcript level (but not protein) level. An advance in *per *expression coupled with temperature sensitive splicing at the 3' UTR of the gene is thought to enable the fly to seasonally adapt to cold, short days perhaps by increasing activity levels earlier in the day to combat early nightfall [29,30]. Seasonal variation in behavioral rhythms (free running period or FRP) exist in other organisms, including honeybees from colonies sampled in different months of the year [31]. Harvester ant colonies experience a seasonal dormancy during the colder months of year, with very low or no external foraging activity outside of the nests. In this study, we see an advance in the timing of daily *per *upregulation in foragers collected in early spring (cold, short days) relative to foragers collected in early fall (long, warm days). Thus, our results are consistent with results from studies of seasonal variation in per expression patterns in fruitflies [29,30]. The advance in timing of high *per *expression levels in harvester ants in the spring relative to early fall may function to increase the activity of foragers earlier in the day when more light is available and temperatures are less extreme. Further work will elucidate whether the advance in *per *expression is directly related to seasonal behavioral patterns of foragers in field colonies and what mechanism drives the shift in the expression cycle of *per *mRNA in ants.

## Conclusion

The circadian clock pathway has emerged as an intriguing potential mechanism involved in the division of labor in social insects. The conservation of circadian clock functions across diverse taxa contrasts sharply with new evidence of the surprising degree of divergence in the underlying molecular pathways, especially in insects [32,33]. Particularly exciting is the recent discovery that features of honeybee circadian clocks resemble mammalian clocks more than *Drosophila *clocks [33]. Given the potential diversity of pathways, the association of diurnal or nocturnal locomotor activity with the cyclical expression pattern of the *period *gene has remained one of the most conserved components of the circadian mechanism. Our results and future studies will help elucidate how the ancient system of molecular clock function has been co-opted for similar functions in social insects, providing a fascinating link between chronobiology and sociobiology.

## Methods

### Ants and sampling

Laboratory colonies of harvester ants (*Pogonomyrmex occidentalis*) were established by collecting field colonies in Hurricane, Utah in February/March 2006 (S06, 4 colonies) and September 2006 (F06, 4 colonies). Colonies were transferred to individual plexiglass nest boxes (15 cm wide × 30 cm long × 10 cm high) connected to open-air foraging arenas (25 cm W × 60 cm L × 15 cm H) by short Tygon tubing (2 cm diameter × 10 cm long). Nest box floors had a 3 cm layer of plaster with built-in irrigation system to keep colonies moist. Nest boxes were covered in double layers of red cellophane and had removable lids for ease of observation and collection. Colonies were kept under stable conditions of light, temperature and humidity in controlled environmental chambers (lights on in LD conditions at 6 AM). Laboratory colonies did not contain queens, but did contain some larvae. For both spring and fall experiments, original colonies collected in the field were divided into two queenless laboratory colonies of at least 400 workers that were then subjected to the different entrainment regimes. In the S06 study, each of four colonies were divided into two laboratory colonies and entrained in either 12 h:12 h light-dark (LD) conditions (4 colonies) or 24 h dark-dark (DD) conditions (4 colonies) at 15/12.5°C with ~70% relative humidity. Collection of S06 time point samples occurred in DD conditions at a constant temperature of 14.5°C. In the F06 study, each of five colonies were divided into two laboratory colonies and entrained in either 12 h:12 h light-dark (LD) conditions (5 colonies) or 24 h dark (DD) conditions (3 colonies only) at 19/16°C with ~80% relative humidity. Collection of F06 time point samples occurred in DD conditions at constant temperature of 19°C.

Foragers were identified as ants that were observed on the food in the foraging arena and were labeled using a dot of acrylic paint during the day. Nest workers were identified as ants that remained in the covered nest box and were never observed in the foraging arena. Exact ages of the foragers and nest workers were not known, but nest workers are typically younger than foragers [23,34,35]. We expect there may be seasonal differences in the age of nest workers – in the spring, a burst of brood production [34] yields nest workers that are likely to be younger than their fall counterparts. We do not expect seasonal differences in the ages of foragers. Thus, comparisons between nest workers and foragers were conducted in the spring.

We collected multiple ants (6–10) at each timepoint and reported sample sizes are the RNA samples successfully extracted from these collections (either 3 or 4 individuals per colony and 3–5 colonies per time point, depending on season). In the S06 study, RNA samples from three ants were collected from each colony per time point of collection; in the F06 study, RNA samples from four ants were collected from each colony. All sampling was done using dim red light in dark conditions. Ants were collected from each colony at six time points: 4:00, 8:00, 12:00, 16:00, 20:00, and 24:00 hours. Individual ants were immediately immersed in liquid nitrogen and stored at -70°C until dissection. Brain dissections were performed in 50 uL 1× PBS and 5 uL RNAlater^® ^under a dissecting microscope. RNA isolation was done using the RNeasy Plus Mini Kit according to manufacturer's instructions (Qiagen) and isolated RNA was stored at -70°C. In the spring study, RNA was purified from 144 individual brain dissections, 432 cDNA reactions were amplified and 864 qPCR reactions were analyzed for the two genes. In the fall study, RNA was purified from 192 individual brain dissections, cDNA was amplified in triplicate for each RNA sample (n = 576) and 1152 qPCR reactions were analyzed.

### Quantitative real-time PCR

Conserved blocks of amino acid sequence from known *period *genes in *Drosophila melanogaster*, *Apis mellifera*, *Bombus ardens*, and *Formica japonica *were identified with the CODEHOP program [36,37]. Degenerate primers (see Additional file 1) were designed to amplify the ant ortholog to *period *(ABI Big-Dye Sequencing technology on an ABI 377 instrument) from genomic DNA and cDNA. Harvester ant-specific primers were designed from exon-coding regions to amplify a 90 bp region for qPCR analyses. Percent nucleotide/amino acid similarity to known eusocial insect *per *sequences was estimated using MacVector^® ^(*F. japonica*, 92/93%; *A. mellifera*, 76/82%; *B. ardens*, 74/78%). Real time primers (PoPerF: 5'-TCCTTCAGGTCGAAGCCGT-3'; PoPerR:5'-TGATAAAGGACGACCACTCGG-3') and Taqman probe^® ^(PoPerT:5'DFAM-CAGATTCGCCGTGCAGAACGGG-3'DTAM) were designed using Primer Express^® ^software (ABI).

cDNA was synthesized from extracted total RNA preps using ABI TaqMan Reverse Transcriptase reagents and an oligo-dT primer. Reactions were performed in triplicate for each individual brain. All reactions were run at 25°C for 10 minutes, 48°C for 30 minutes, 95°C for 5 minutes, and then stored at -80°C until quantitative PCR. For each cDNA replicate, expression of *PoPer *was assayed on an ABI 7900 HT instrument using ABI Taqman Gold reagents. To standardize *period *expression, an ant homolog of the RNA polymerase II 512 kD (*PoRPII*) subunit was used as a control [38] for each cDNA replicate (see Additional file 1). Real-time PCR reactions for *Poper *and *PoRPII *were performed under the following conditions: 2 min 50°C, 10 min 95°C, 15 sec 95°C, 1 min 58°C, for 45 cycles. Data was analyzed using SDS 2.1 software and quantification of relative mRNA levels was calculated using the ΔΔCt method. Three (S06) or four (F06) individual brains were pooled to calculate a colony value per time point. Colony values were averaged at each time point for comparisons across a 24 hr period. Differences in relative levels of *PoPer *expression over time were analyzed for each task and condition using one-way analysis of variance tests. Differences in expression levels between foragers in LD and DD conditions (S06 & F06) and between foragers and nest workers (S06 only) were analyzed using two-way analysis of variance tests. Differences between colonies (S06 & F06) were analyzed using repeated measures analysis of variance tests. All analyses were performed using SPSS software.

### Analysis of *P. occidentalis *locomotor activity

We observed daily activity patterns of individual nest workers and foragers from colonies of *P. occidentalis *in the absence of social interactions and light stimulus. Colonies were collected in April and entrained for five days in 12:12 LD conditions. Individual nest workers (n = 6) and foragers (n = 5) were removed from colonies and placed in Petri dishes with a simple line bisecting the bottom of the dish. Ants were placed in separate environmental chambers under constant temperature (18°C) and humidity (80%) conditions with a DD light regime for 24 hours. On the second day in DD conditions, ants were videotaped using infrared technology (Sony Handycam) over 24 hour periods and activity was measured via manual inspection as the number of times an individual crossed the line per hour by observers who were blind to the experimental conditions. Activity levels of individuals were averaged over two-hour intervals for each task (Figure 4). In addition, four-hour 'bins' were constructed to correspond to the six time points in the RNA analyses by averaging values per individual (eg. 6 AM-10 AM values represented the 8 AM time point). Differences between nest worker and forager daily activity patterns over the six time points were analyzed with a two-way repeated measures ANOVA.

## Authors' contributions

KI conceived of the study, participated in molecular genetic work and statistical analyses, and drafted the manuscript. SK and ML participated in the design of the study, the molecular genetic work and statistical analyses. All authors read and approved the final manuscript.

## Supplementary Material

Additional file 1 **Degenerate cloning primers and procedure**. This file outlines the procedure for obtaining PoPer and RPII clones from *P. occidentalis *and includes the degenerate and real-time primers used in the study. Click here for file
