# Supplementary material for: Expression patterns of a circadian clock gene are associated with age-related polyethism in harvester ants, Pogonomyrmex occidentalis
Source: BMC Ecol. 2009 Apr 17;9:7. doi: 10.1186/1472-6785-9-7 (PMC2676274; doi:10.1186/1472-6785-9-7)
Supplement: Additional file 1 — Degenerate cloning primers and procedure. This file outlines the procedure for obtaining PoPer and RPII clones from P. occidentalis and includes the degenerate and real-time primers used in the study. [file 1472-6785-9-7-S1.pdf]

Additional File 1:

Degenerate primers for cloning of *PoPer:*

**Primer Name**	**Sequence (5'-3')**	**Corresponding Conserved AA sequence**
MBF03_F	CTGCGTGATCTCCatgcaygaygg	CCVISMHDGL
GEF01_F	CCGTCCTACAACCAGCTGaaytayaayga	PSYNQLNYNE
GER02_R	GAAGAAGCGCTCGATGTTCtcrttrtartt	YNENIERFFK
MIR06_R	CGGTGCTTCTTCATCATCAACttytccatntc	DMEKLMMLKHR

Cloning procedure and real time qPCR primers:

Conserved blocks of amino acid sequence from known *period* genes in *Drosophila melanogaster*, *Apis mellifera*, *Bombus ardens*, and *Formica japonica* were identified with the CODEHOP program [36, 37]. Amplification of products from genomic DNA with CODEHOP degenerative primers was achieved using the standard protocol described in [36, 37] using Qiagen® PCR Core Kit reagents. Products of correct size were purified using Qiaquick PCR Clean-Up kits (Qiagen®) and cloned using a TOPO TA cloning kit (Invitrogen®). Clones were re-amplified in a new PCR reaction, purified with Sephadex® G50 spin columns and sequenced using an ABI 377 instrument with Big-Dye chemistry (ABI). Internal primers specific to *P. occidentalis* were designed and cDNA was amplified using these specific primers. Harvester ant-specific primers were designed from exon-coding regions to amplify a 90bp region for qPCR analyses. Clone sequences for PoRPII (endogenous control gene) and PoPer can be accessed on Genbank (Accession # FJ853425 & FJ853426). Real time primers (PoPerF: 5'-TCCTTCAGGTCGAAGCCGT-3'; PoPerR:5'-TGATAAAGGACGACCACTCGG-3') and Taqman® probe (PoPerT:5'DFAM-CAGATTCGCCGTGCAGAACGGG-3'DTAM) were designed using Primer Express® software (ABI). The endogenous control gene, RNA Polymerase II subunit 215 (*RPII*), was amplified with harvester ant *RPII*-specific forward and reverse primers (Forward: 5'-GAGAACCAAGTGAACAGGAT-3', Reverse: 5'-TTATTGTATTCAGTCAGGGATTTC-3') and TaqMan probe (5'DFAM-AGAGCCTCCAGTCTTGTCTCGA-3'DTAM).
